# Revelation of the ability of *Burkholderi*a sp. USM (JCM 15050) PHA synthase to polymerize 4-hydroxybutyrate monomer

**DOI:** 10.1186/2191-0855-2-41

**Published:** 2012-08-09

**Authors:** Nyok-Sean Lau, Kumar Sudesh

**Affiliations:** 1 Ecobiomaterial Research Laboratory, School of Biological Sciences, Universiti Sains Malaysia, Penang, 11800, Malaysia

**Keywords:** Biopolymer, Polyhydroxyalkanoate, PHA synthase, PHA operon, *Burkholderia* sp.

## Abstract

The nutrition-versatility of *Burkholderia* sp. strain USM (JCM 15050) has initiated the studies on the use of this bacterium for polyhydroxyalkanoate (PHA) production. To date, the *Burkholderia* sp. has been reported to synthesize 3-hydroxybutyrate, 3-hydroxyvalerate and 3-hydroxy-4-methylvalerate monomers. In this study, the PHA biosynthetic genes of this strain were successfully cloned and characterized. The PHA biosynthetic cluster of this strain consisted of a PHA synthase (*phaC*), β-ketothiolase (*phaA*), acetoacetyl-CoA reductase (*phaB*) and PHA synthesis regulator (*phaR*). The translated products of these genes revealed identities to corresponding proteins of *Burkholderia vietnamiensis* (99–100 %) and *Cupriavidus necator* H16 (63–89%). Heterologous expression of *phaC*_*Bs*_ conferred PHA synthesis to the PHA-negative *Cupriavidus necator* PHB¯4, confirming that *phaC*_*Bs*_ encoded functionally active protein. PHA synthase activity measurements revealed that the crude extracts of *C. necator* PHB¯4 transformant showed higher synthase activity (243 U/g) compared to that of wild-types *Burkholderia* sp. (151 U/g) and *C. necator* H16 (180 U/g). Interestingly, the transformant *C*. *necator* PHB¯4 harbouring *Burkholderia* sp. PHA synthase gene accumulated poly(3-hydroxybutyrate-*co*-4-hydroxybutyrate) with 4-hydroxybutyrate monomer as high as up to 87 mol% from sodium 4-hydroxybutyrate. The wild type *Burkholderia* sp. did not have the ability to produce this copolymer.

## Introduction

Since the introduction of phenoformaldehyde plastic in 1909 by Leo Hendrik Baekeland, petrochemical plastics have developed into a major industry and an indispensable commodity for modern life (Meikle, [1995]). It is estimated that more than 100 million tonnes of plastics are produced yearly. Most of these plastics end up after their useful life as discarded waste and some are disposed into the marine environment which pose a threat to the aquatic wildlife. In recent years, the widespread and increasing use of petrochemical plastics has raised concerns about the adverse impact of these recalcitrant plastics on the environment. Hence, biobased and biodegradable polymers are gaining widespread interest and acceptance as an alternative to some synthetic plastics. Polyhydroxyalkanoate (PHA), a storage and reserve compound accumulated naturally in the cytoplasm of numerous bacteria, is being considered as one of the most attractive and promising biodegradable thermoplastics for various industrial and biomedical applications (Sudesh and Iwata, [2008]).

Poly(3-hydroxybutyrate) [P(3HB)], the most common type of PHA, has mechanical properties such as Young’s modulus and tensile strength similar to polypropylene. Nevertheless, P(3HB) is a stiffer and more brittle plastic material compared to polypropylene (Tsuge, [2002]). Many other types of PHA with improved mechanical properties are synthesized by incorporating co-monomers such as 3-hydroxyvalerate, 3-hydroxyhexanoate and 4-hydroxybutyrate. The underlying challenge for the commercialization of PHA is the higher production cost compared to petrochemical plastics. To achieve the commercial application and wide use of PHA, efforts are directed on effectively lowering the production cost of PHA. Much research has been focused on reducing the production cost by strain development, developing more efficient fermentation and recovery processes and using inexpensive carbon sources. At the same time, more PHA applications are being developed, including the high value applications such as medical and pharmaceutical field at which cost of production is not the main concern (Chen, [2009]).

Structural analyses in recent years revealed that different types of granule-associated proteins are located on the surface of PHA granules. The proteins include PHA synthases, intracellular PHA depolymerases, phasins, PHA synthesis regulator proteins and etc. (Pötter and Steinbüchel, [2006]; Rehm, [2006]; Jendrossek, [2009]). PHA synthases are the most important enzymes involved in PHA biosynthesis. They can be grouped into four classes based on their *in vivo* substrate specificities, primary amino acid sequences and subunit composition (Rehm, [2007]). Class І synthases, which are represented by the PHA synthase from *Cupriavidus necator,* are active towards short-chain length (*R*)-hydroxyacyl-CoA consisting of three to five carbon atoms (Pötter and Steinbüchel, [2006]). Class ІІ synthases are active towards medium-chain length (*R*)-3-hydroxyacyl-CoA that contain six to fourteen carbon atoms and are represented by *Pseudomonas aeruginosa*. Class І and ІІ PHA synthases comprise enzymes consisting of only one type of subunit (PhaC) with molecular masses (Mw) between 61 and 68 kDa. Class ІІІ PHA synthases, represented by the *Allochromatium vinosum* PHA synthases, comprise two subunits: PhaC and PhaE. Class IV PHA synthases, represented by the enzyme of *Bacillus megaterium*, consist of two different types of subunits (PhaC and PhaR). Both class ІІІ and IV PHA synthases prefer short-chain length (*R*)-hydroxyacyl-CoA. (Rehm, [2003]).

*Burkholderia* sp. USM (JCM 15050) was isolated from oil-polluted wastewater and has been known to utilize various carbon sources e.g. sugars, organic acids and triglycerides for PHA production (Chee et al. [2010]; Lau et al. [2010]). The focus of this research was the PHA biosynthetic genes cloned from this bacterium. Here, we reported for the first time that the heterologous expression of *Burkholderia* sp. PHA synthase in *C*. *necator* PHB¯4 lead to the accumulation of poly(3-hydroxybutyrate-*co*-4-hydroxybutyrate), P(3HB-*co*-4HB) with high 4-hydroxybutyrate (4HB) composition. Although the accumulation of PHA containing 4HB monomer was not observed in wild-type *Burkholderia* sp. culture, the PHA synthase of *Burkholderia* sp. was able to polymerize the 4HB monomer.

## Materials and methods

### Bacterial strains, plasmids and media

All bacterial strains and plasmids used in this study are listed in Table 1. *C*. *necator* PHB¯4 was cultivated in nutrient rich (NR) medium containing meat extract (10 g/L), peptone (10 g/L) and yeast extract (2 g/L) at 30°C. *Escherichia coli* strains were grown at 37°C in Lysogeny Broth (LB) medium consisting of the following components (per L): 10 g casein enzyme hydrolysate, 5 g yeast extract and 10 g NaCl. For maintenance of plasmid, kanamycin (50 μg/mL) or ampicilin (100 μg/mL) were added.

**Table 1:** Bacterial strains and plasmids used in this study

**Strain or plasmid**	**Relevant characteristics**	**Source or reference**
**Strains**		
*E*. *coli* JM109	*recA1*, *endA1*, *gyrA96*, *thi*, *hsdR17*, *supE44*, *relA1*, Δ(*lac-proAB*)/F'[*traD36*, *proAB*^+^, *lacI*^q^, *lacZ*ΔM15]	Promega
*E*. *coli* S17−1	*relA1*, Δ*lacU169*, (ϕ80*lacZ*ΔM15), *thi1*, *proA*, *hsdR17*, *hsdM*^+^, *recA*, RP4-tra function	Simon et al., [1983]
*C*. *necator* PHB¯4	PHA-negative mutant of wild-type H16	Schlegel et al., [1970]
*Burkholderia* sp. USM (JCM 15050)	Wild type	Chee et al., [2010]
		
**Plasmids**		
pGEM-T	Ap^r^, *lacZ*, cloning vector	Promega
pBBR1MCS-2	Km^r^, l*acPOZ*' *mob*^+^, broad host range	Kovach et al., [1995]
pBBR1MCS-2 *phaC*_*Bs*_BH	pBBR1MCS-2 derivative harbouring *Bam*HI-*Hind*III *phaC* from *Burkholderia* sp. with putative promoter	This study
pBBR1MCS-2 *phaC*_*Bs*_BE	pBBR1MCS-2 derivative harbouring *Bam*HI-*Eco*RI *phaC* from *Burkholderia* sp. with putative promoter	This study

### Culture conditions for the synthesis of PHA

To determine the functional expression of the cloned PHA biosynthetic genes *in vivo*, PHA biosynthesis was carried out with transformant *C*. *necator*. Both one-stage cultivation and two-stage cultivation were carried out according to the method described previously (Lau et al., [2010]; Lau et al., [2011]). For inoculum preparation, transformant *C*. *necator* was grown in 50 mL of NR at 30°C, 200 rpm. 3% (v/v) of the total volume of the seed culture (OD_600 nm_ = 4.5) were inoculated into nitrogen-limiting mineral salts medium (MM). The MM contained (per liter): 3.32 g Na_2_HPO_4_, 2.80 g KH_2_PO_4_, 0.50 g NH_4_Cl, 0.25 g MgSO_4_·7H_2_O and 1 mL trace elements solution (Doi et al., [1995]). The trace element solution consisted of 0.22 g CoCl_2_·6H_2_O, 9.7 g FeCl_3_, 7.8 g CaCl_2_, 0.12 g NiCl_2_·6H_2_O, 0.11 g CrCl_3_·6H_2_O, 0.16 g CuSO_4_·5H_2_O in one liter 0.1 N HCl (Kahar et al., [2004]). Different carbon sources (crude palm kernel oil, jatropha oil, fructose, 4-methylvaleric acid, sodium propionate, sodium valerate, γ-butyrolactone and sodium 4-hydroxybutyrate) were tested for their ability to promote PHA synthesis in transformant *C*. *necator*. The cultures were harvested by centrifugation (6000 *g*, 7 min and 4°C) after 48 h of incubation at 200 rpm, 30°C. Cell pellets were washed with hexane to get rid of excess oil-based carbon sources before being washed with distilled water. Cells grown in non-palm oil based carbon sources were washed only with distilled water.

For two-stage cultivation, 3% (v/v) of the inoculum from transformant *C*. *necator* culture was transferred into fresh NR broth which was then incubated for additional 24 h. The culture was centrifuged aseptically (6000 *g*, 7 min and 4°C) and washed with sterile distilled water before being transferred into nitrogen-free MM. The cultures were incubated at 30°C, 200 rpm for 48 h before being harvested.

### Analytical procedures

The PHA content and composition were analyzed by gas chromatography (GC) spectrometry. Approximately 25 mg of lyophilized cells were subjected to methanolysis in the presence of 15%(v/v) sulphuric acid in methanol. The resulting hydroxyacyl methyl esters were assayed according to the method of Braunegg and co-workers. (Braunegg et al., [1978]).

### DNA manipulation

The isolation of genomic DNA from *Burkholderia* sp., plasmids DNA isolation, agarose gel electrophoresis and transformation of *E*. *coli* were performed following standard procedures (Sambrook et al., [1989]). Restriction endonucleases, all other DNA-manipulating enzymes and kits were used as recommended by the manufacturers (Promega, USA).

### Cloning of PHA biosynthetic genes from *Burkholderia* sp

The putative PHA synthase (*phaC*_*Bs*_), β-ketothiolase (*phaA*_*Bs*_), acetoacetyl-CoA redutase (*phaB*_*Bs*_) and PHA synthesis regulator (*phaR*_*Bs*_) genes were amplified form the chromosomal DNA of *Burkholderia* sp. using primer pairs І, ІІ and ІІІ respectively (Table 2). The resulting polymerase chain reaction (PCR) products were cloned into pGEM-T vector (Promega, USA) and sent for sequencing at 1st BASE Laboratory (Malaysia). Sequence comparisons and alignments were performed with the Basic Local Alignment Search tool (BLAST, National Center for Biotechnology Information) and ClustalW Multiple Sequence Alignment program (Thompson et al., [1994]). The potential promoter regions recognized by Sigma factor D (σ^D^) were predicted using prediction of bacterial promoters (BPROM) provided by Softberry Inc. (http://www.softberry.com).

**Table 2:** **List of primers used in this study**^**a**^

**Primer pairs**	**Primers’ sequences**	**Target gene**
І	F: GCGAGTCACCGAAAATGTTTTATGTT	*Burkholderia* sp. *phaC* gene
	R: TGCGGCCCCTTCAGGTAGTTGTC	
ІІ	F: AAGAAGAAGCGCAGCTACTGGGTC	*Burkholderia* sp. *phaA* gene
	R: TTGAACAGGCTCGTCAGATTGGTG	
ІІІ	F: GGCCAGACCAACTATTCGACCGC	*Burkholderia* sp. *phaB* and *phaR* genes
	R: GCGACGGCCTTACTTCTTTTCCG	
IV	F: GCGAGGATCCGAAAATGTTTTATGTT	*Burkholderia* sp. *phaC *gene
	R: ATGAGCTCACTACGTCCGTCATTTCC	
V	F: GCGAGGATCCGAAAATGTTTTATGTT	*Burkholderia* sp. *phaC* gene
	R: ATGAATTCACTACGTCCGTCATTTCC	

^a^ All primers were synthesized by 1st BASE Laboratory (Malaysia). Restriction enzymes digestion sites were underlined.

### Construction of plasmids

To construct a plasmid for expression of *phaC*_*Bs*_ in *C*. *necator* PHB¯4, PCR was performed with primer pairs IV to obtain the gene fragment containing the putative coding region, ribosome binding site and promoter. This PCR product was digested with *BamH*І and *Sac*І and inserted in frame into *BamH*І- and *Sac*І- restricted pBBR1MCS-2. The *phaC*_*Bs*_ was also cloned in reverse orientation to *lac* promoter in pBBR1MCS-2 to confirm the expression under its native promoter. The plasmid for expression was constructed by ligation of *phaC*_*Bs*_ amplified with primer pairs V into pBBR1MCS-2 at *BamH*І and *EcoR*І sites. Conjugation of *C*. *necator* PHB¯4 with *E*. *coli* S17–1 harbouring broad-host-range plasmids were performed as described by Friedrich et al., [1981].

### PHA synthase activity assay

Wild-type *Burkholderia* sp. and transformant *C*. *necator* harbouring pBBR1MCS-2 *phaC*_*Bs*_BH were cultivated for 24 h under conditions as described in the materials and methods section. Cells were harvested and resuspended in 20 mM Tris-HCl (pH 8). Cell extracts were obtained by disruption using sonication (two cycles, 7 min each) with a TOMY UD-200 sonicator and centrifugation at 13700 x *g* for 10 min at 4°C. Activities of PHA synthase were determined spectrophotometrically by monitoring the release of CoA at 412 nm (30°C). The standard assay contained 40 mM potassium phosphate buffer (pH 7.5), 2 mM 3HB-CoA, 10 mM 5,5’-dithio-bis(2-nitrobenzoic acid) and 35 to 40 μg of protein from soluble protein fraction (Bhubalan et al. [2011]). One unit of enzyme activity was defined as the amount of enzyme that catalyzed the release of 1.0 μmol CoA/min using a molar absorption coefficient of 13,600 M^−1^ cm^−1^. The protein concentration was assayed as described by Bradford using bovine serum albumin as standard.

## Results

### Identification and cloning of PHA biosynthetic genes from *Burkholderi*a sp. USM (JCM 15050)

**Figure 1:**
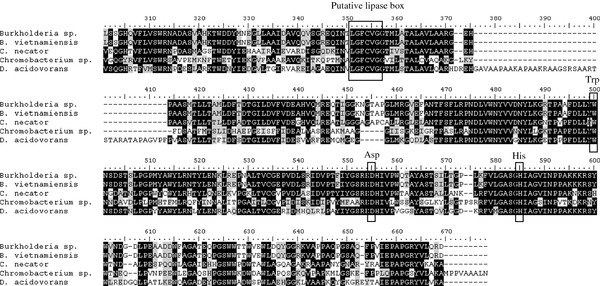
**Multiple alignment of the partial deduced amino sequences of *phaC* of *Burkholderia* sp**. USM (JCM 15050) with corresponding *phaC* sequence from *Burkholderia vietnaminesis* G4 (Genbank accession no. YP_001119557.1), *Burkholderia* sp. DSMZ9242 (GenBank accession no. AAF23364.1), *Cupriavidus necator* H16 (GenBank accession no. YP725940.1), *Chromobacterium* sp. USM2 (Genbank accession no. ADL70203.1) and *Delftia acidovorans* (Genbank accession no. BAA33155.1).

In order to determine the nucleotide sequence of PHA locus of *Burkholderia* sp. USM (JCM15050), primers for amplification were designed based on the annotated *phaC*, *phaA*, *phaB* and *phaR* obtained from genomic DNA sequencing of *Burkholderia vietnamiensis*, *Burkholderia thailandensis*, *Burkholderia ambifaria*, *Burkholderia glumae*, *Burkholderia mallei*, *Burkholderia multivorans* and *Burkholderia cenocepacia*. The nucleotide sequence obtained were deposited with GenBank (accession no. JN022533, JN835296-JN835297, JQ936592). The deduced amino acid sequence of *phaC*_*Bs*_ exhibited great similarity to PhaC of *B*. *vietnamiensis* (99% identity, accession no. YP_001119557.1), *Pseudomonas putida* (73% identity, accession no. BAB96552.1) and *Cupriavidus necator* (63% identity, accession no. YP_725940.1). The putative Shine-Dalgarno consensus sequences (TAAGG) were found 10 base pairs upstream of the putative start codon for *phaC*_*Bs*_. The putative −35 region (TTCACA) and −10 region (TGATAAAAA) found were similar to the corresponding sequences of *E*. *coli* σ70 consensus promoter sequence. The product of *phaC*_*Bs*_ is a protein composed of 625 amino acids with a calculated molecular mass of 68.25 kDa. Although the nucleotide sequence of *phaC* from *B. vietnamiensis* was available following complete genomic DNA sequencing, functional characterization of the gene has not been reported. Comparison of the deduced amino acids sequence of *phaC* from *Burkholderia* sp. with homologous *phaC* genes from other bacteria was done by multiple alignment (Figure 1). The deduced amino acids sequence of *phaC*_*Bs*_ showed relatively high identity to other PHA synthase and the putative lipase box that has been found conserved in all the PHA synthase was also identified. The deduced amino acid sequences of putative *phaA*_*Bs*_ were similar to those β-ketothiolase from *B*. *vietnamiensis* (99% identity, accession no. YP_001119556.1), *P. putida* (90% identity, accession no. BAB96553.1) and *C. necator* (87% identity, accession no. YP_725941.1). The putative *phaB*_*Bs*_ exhibited significant identity to acetoacetyl-CoA redutase from *B*. *vietnamiensis* (99% identity, accession no. YP_001119555.1), *P. putida* (92% identity, accession no. BAB96554.1) and *C. necator* (89% identity, accession no. YP_725942.1). A putative PHA synthesis regulator is encoded by *phaR*_*Bs*_ and it is located downstream of *phaB*. The deduced amino acid sequence of *phaR* exhibited high identity to *B*. *vietnamiensis* (100% identity, accession no. YP_001119554.1) and *C. necator* (83% identity, accession no. YP_725943.1). As *Burkholderia* sp. 16 S rDNA gene (accession no. FJ667272.1) exhibited 92% identity to the corresponding gene of *C*. *necator* (accession no. CP000090.1), it is not surprising to find that the translated products of *Burkholderia* sp. PHA biosynthetic genes revealed identities to corresponding proteins of *C. necator* (63–89%).

### PHA accumulation in transformant *C*. *necator* PHB¯4

The functionality of the cloned *Burkholderia* sp. USM (JCM 15050) PHA synthase gene was investigated by heterologous expression of the gene in the PHA-negative *C*. *necator* host. Plasmid pBBR1MCS-2 *phaC*_*Bs*_BH containing *phaC*_*Bs*_ and its putative promoter collinear to and downstream of the *lac* promoter was constructed. The expression was confirmed by complementation of the mutant host deficiency in PHA synthesis and the PHA-producing ability of the transformant strains were examined (Table 3). Interestingly, the heterologous expression of *phaC*_*Bs*_ in *C*. *necator* PHB¯4 has conferred the ability to synthesize P(3HB-*co*-4HB) copolymer with high 4HB composition in the mutant host strain. The 4HB composition reached 87 mol% in cultures supplemented with sodium 4-hydroxybutyrate in two-stage cultivation. In contrast, wild type *Burkholderia* sp. has limited ability to utilize 4HB-related carbon sources for growth and PHA production (results not shown). The highest PHA content of 66 wt% accompanied by relatively high dry cell weight of 2.4 g/L were obtained in transformant *C*. *necator* harbouring *phaC*_*Bs*_ cultivated on crude palm kernel oil (CPKO) in one-stage cultivation. Although a low fraction of 3-hydroxy-4-methylvalerate (3H4MV) monomer was synthesized by the transformant strain culture from 4-methylvaleric acid, it is in agreement with previous studies on the ability of *Burkholderia* sp. PhaC to polymerize 3H4MV monomer (Lau et al., [2010]). 3-hydroxyvalerate (3HV) monomer was also incorporated into the polymer in the presence of precursor carbon sources e.g. sodium valerate and sodium propionate. To examine whether the expression of *phaC*_*Bs*_ was affected by the *lac* promoter, pBBR1MCS-2 *phaC*_*Bs*_BE was constructed by inserting *phaC*_*Bs*_ in reverse orientation to *lac* promoter. Transformant *C*. *necator* PHB¯4 harbouring pBBR1MCS-2 *phaC*_*Bs*_BE showed similar cell growth and PHA accumulation to strain harbouring pBBR1MCS-2 *phaC*_*Bs*_BH. These results suggest that the *Burkholderia* sp. PHA synthase gene was constitutively expressed in C. *necator* PHB¯4 from the native promoter.

**Table 3:** **Effect of different carbon sources on the biosynthesis of PHA by transformant *Cupriavidus necator* PHB¯4 harboring the PHA synthase gene of *Burkholderia* sp.**

**Carbon sources**^**a**^	**Dry Cell Weight**^**b**^**(g/L)**	**PHA content**^**c**^**(wt %)**	**PHA composition**^**d**^**(mol %)**			
			**3HB**	**3HV**	**3H4MV**	**4HB**
One-stage cultivation						
**pBBR1MCS-2 *phaC* **_** *Bs* **_**BE**						
CPKO	2.1 ± 0.4	64 ± 9	100	0	0	0
**pBBR1MCS-2 *phaC* **_** *Bs* **_**BH**						
CPKO	2.4 ± 0.1	66 ± 2	100	0	0	0
Jatropha oil	2.3 ± 0.1	53 ± 1	100	0	0	0
Fructose	2.5 ± 0.1	61 ± 1	100	0	0	0
Fructose+ 4MV	1.6 ± 0.2	40 ± 8	97	0	3	0
Two-stage cultivation						
Sodium propionate	2.7 ± 0.1	27 ± 3	70	30	0	0
Sodium valerate	3.2 ± 0.1	35 ± 1	60	40	0	0
γ-butyrolactone	2.3 ± 0.1	29 ± 1	69	0	0	31
sodium 4-hydroxybutyrate	2.0 ± 0.1	14 ± 2	13	0	0	87

CPKO, crude palm kernel oil; 4MV, 4-methylvaleric acid; 3HB, 3-hydroxybutyrate; 3HV, 3-hydroxyvalerate 3H4MV, 3-hydroxy-4-methylvalerate; 4HB, 4-hydroxybutyrate.

^a^Cells were cultivated for 48 h, at 30 °C, 200 rpm in MM medium containing the indicated carbon sources (0.2 M carbon concentration: CPKO, jatropha oil, fructose, sodium propionate, sodium valerate, γ-butyrolactone, sodium 4-hydroxybutyrate and 0.02 M carbon concentration: 4-methylvaleric acid).

^b^Dry cell weight after freeze-drying.

^d^PHA composition of the freeze-dried cells was determined by gas chromatography.

The ability of transformant *C*. *necator* PHB¯4 harbouring *Burkholderia* sp. PHA synthase gene to incorporate 4HB monomer into the PHA produced was investigated. To study the effect of 4HB-related carbon sources efficiency and concentration on 4HB composition of PHA synthesized, shake-flask cultures of transformant *C*. *necator* were cultivated on medium with different concentrations of γ-butyrolactone or sodium 4-hydroxybutyrate in two-stage cultivation. The dry cell weight, PHA content and 4HB composition showed slight increase with the increase of sodium 4-hydroxybutyrate concentration from 0.1 to 0.2 M carbon concentration for transformant *C*. *necator* cultures (Table 4). A maximum of 87 mol% 4HB composition was produced by the transformant strain cultivated on medium supplemented with 0.2 M carbon concentration sodium 4-hydroxybutyrate. The increase of sodium 4-hydroxybutyrate concentration from 0.2 to 0.4 M carbon concentration resulted in the decrease of 4HB composition. On the other hand, increased mol fractions of 4HB monomers in PHA produced were achieved by increasing γ-butyrolactone concentrations.

**Table 4:** **Effect of different 4HB-related carbon sources concentrations on the biosynthesis of PHA by transformant *Cupriavidus necator* PHB¯4 harboring the PHA synthase gene of *Burkholderia* sp.**

**Carbon sources**^**a**^	**Carbon concentration (M)**	**Dry Cell Weight**^**b**^**(g/L)**	**PHA content**^**c**^**(wt %)**	**PHA composition**^**d**^**(mol %)**	
				**3HB**	**4HB**
γ-butyrolactone	0.1	1.9 ± 0.1	10 ± 1	78	22
γ-butyrolactone	0.2	2.3 ± 0.1	29 ± 1	69	31
γ-butyrolactone	0.3	2.4 ± 0.2	31 ± 3	68	32
γ-butyrolactone	0.4	2.2 ± 0.1	24 ± 1	59	41
sodium 4-hydroxybutyrate	0.1	1.5 ± 0.1	Trace	28	72
sodium 4-hydroxybutyrate	0.2	2.0 ± 0.1	14 ± 2	13	87
sodium 4-hydroxybutyrate	0.3	1.9 ± 0.1	8 ± 1	40	60
sodium 4-hydroxybutyrate	0.4	2.0 ± 0.1	11 ± 2	46	54

3HB, 3-hydroxybutyrate; 4HB, 4-hydroxybutyrate.

^a^Cells were cultivated for 48 h, at 30 °C, 200 rpm in MM medium containing different concentrations of 4HB-related carbon sources at two-stage cultivation.

^b^Dry cell weight after freeze-drying.

^c^PHA content of the freeze-dried cells was determined by gas chromatography.

^d^PHA composition of the freeze-dried cells was determined by gas chromatography.

### Assay of PHA synthase activity

In order to determine and compare the expression of *Burkholderia* sp. PHA synthase gene in *C*. *necator* PHB¯4 and its native host, crude extracts of transformant *C*. *necator* and wild-type *Burkholderia* sp. were subjected to PHA synthase assay (Table 5). The crude lysate from *Burkholderia* sp. exhibited specific activity of 151 U/mg, which was similar and comparable with that from C. *necator* H16 results published previously (180 U/mg) (Schubert et al., [1988]). *C*. *necator* PHB¯4 harbouring *Burkholderia* sp. PHA synthase exerted slightly enhanced *in vivo* PHA synthase activity compared to wild-type *Burkholderia* sp.

**Table 5:** **Analysis of PHA synthase activity in wild-type *Burkholderia* sp. and transformant *C* . *necator* **

**Strain**	**PHA synthase sp act (U/g of protein)**^**a**^	**References**
*Burkholderia* sp. USM (JCM 15050)	151	This study
*C. necator* PHB¯4 (pBBR1MCS-2 *phaC*_*Bs*_BH**)**	243	This study
*C. necator* H16	180	Schubert et al., [1988]

^a^ One unit of enzyme activity was defined as the amount of enzyme that catalyzed the release of 1.0 μmol CoA/min.

## Discussions

**Figure 2:**
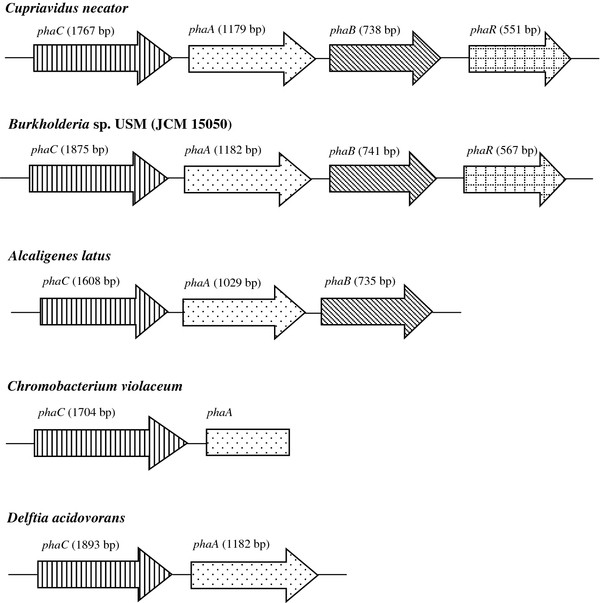
**Molecular organizations of PHA biosynthetic genes in *Burkholderia* sp.** USM (JCM 15050) and other bacteria containing type I PHA synthase (Choi et al., [1998]; Kolibachuk et al., [1999]; Rehm & Steinbüchel, [2002]; Sudesh et al., [1998]).

Bacteria belonging to the genus *Burkholderia* was first used in 1989 for the production of P(3HB) homopolymer from fructose (Ramsay et al., [1989]). They are one of the most nutritionally versatile microorganisms that are capable of utilizing a wide range of carbon sources. In this study, the PHA biosynthetic genes from a locally isolated *Burkholderia* sp. strain USM (JCM 15050) were successfully amplified. The PHA biosynthetic genes of *Burkholderia* sp. consisted of a PHA synthase (*phaC*), β-ketothiolase (*phaA*), acetoacetyl-CoA reductase (*phaB*) and PHA synthesis regulator (*phaR*). The *phaCphaA* and *phaB* seems to be organized in an operon and the structural organization of these genes is closely related to other bacteria harbouring type I PHA synthase e.g. *C*. *necator* and *Alcaligenes latus* (Choi et al., [1998]; Rehm and Steinbüchel, [2002]) (Figure 2). Although the PHA synthase and other PHA biosynthetic genes are often found clustered in the bacterial genomes, there are some exceptions to these observations. In the genomes of *Caulobacter crescentusParacoccus denitrificansMethylobacterium extorquens* and *Aeromonas caviae*, the genes related to PHA biosynthesis are not directly linked to the PHA synthase (Rehm and Steinbüchel, [2002]). Some bacteria e.g. *P*. *denitrificans* contains other genes related to PHA synthesis (phasin, *phaP* and *phaR*) map close to the PHA locus. Both *C*. *necator* and *Burkholderia* sp. PHA locus also possess a putative *phaR* immediately downstream of *phaB*. The *phaR* encodes a protein with putative regulatory function in PHA metabolism (York et al. [2002]; Stubbe and Tian, [2003]; Pötter and Steinbüchel, [2006]).

The putative translated product of *Burkholderia* sp. PHA biosynthetic genes exhibited high homologies to the respective *B*. *vietnamiensis* and *C*. *necator* genes. As the most important enzyme involved in PHA biosynthesis, PHA synthase of *Burkholderia* sp. belongs to Class І that is active on short chain-length (*R)*-hydroxyacyl-CoA with three to five carbon atoms (Rehm and Steinbüchel, [2002]; Rehm, [2007]). Site-specific mutagenesis analysis of *C*. *necator* PHA synthase had shown that the conserved cystein-319, aspartate-480 and histidine-508 of the class I synthase are required for enzyme activity. These amino acid residues are conserved in all class I synthases and it was suggested that they are involved in covalent catalysis. In addition, another conserved residue, tryptophan-398 was suggested to function in PHA synthase subunit dimerization by the provision of a hydrophobic surface (Gerngross et al., [1994]; Jia et al., [2001]). In recent years, PHA synthases with improved substrate specificity, enzyme activity and stability were engineered by various approaches e.g. random mutagenesis, gene shuffling and recombination (Nomura and Taguchi [2007]). The introduction of a mutant PHA synthase (Ser325Thr/Gln481Lys) from *Pseudomonas* sp. 61-3 and lactate (LA) monomer supplying enzymes into *E*. *coli* had enabled the production of LA-based polyesters in a biological system (Shozui et al., [2010]). The same mutant *Pseudomonas* sp. PHA synthase was expressed in *E*. *coli* for the synthesis of glycolate-based polyesters containing medium-chain-length 3-hydroxyalkanoates (Matsumoto et al., [2011]).

The types and compositions of PHA that are produced by the biological system depend on the PHA synthase substrate specificity, the carbon sources supplied and the metabolic pathways that are functioning in the cell (Sudesh and Doi, [2005]). PHA-negative mutant PHB¯4 of *C*. *necator* is a desirable host for PHA production due to the strain ability to achieve stable high cell density fermentation and produce high PHA content from simple, inexpensive substrates (Taguchi et al., [2003]). Heterologous expression of *Burkholderia* sp. PHA synthase gene in PHA-negative mutant of *C*. *necator* enabled the synthesis of P(3HB-*co*-4HB) containing 87 mol% of the 4HB fraction. In contrast, wild-type *Burkholderia* sp. had reduced ability in utilizing 4HB-related carbon sources for growth and PHA production. It is for the first time that the *Burkholderia* sp. PHA synthase was shown to polymerize 4HB monomer. It is well documented that *C*. *necator* was equipped with the metabolic pathway to supply 4HB-CoA substrate even though only small amount of P(4HB) was accumulated (Nakamura et al., [1992]). The expression of *Burkholderia* sp. PHA synthase in a host that are capable of providing 4-hydroxybutyryl-CoA as substrate for the PHA synthase allowed the polymerization of 4HB monomer. These results support previous findings which suggested that the monomer supplying pathways operating in the cells are an important factor determining the provision of substrate for the polymerization by the PHA synthase (Sudesh et al., [1998]). In addition, the synthesis of P(3HB-*co*-4HB) with high 4HB composition by transformant *C*. *necator* suggested the preference of *Burkholderia* sp. PHA synthase for 4-hydroxybutyryl-CoA.

In this study, the PHA biosynthetic gene cluster *Burkholderia* sp. USM (JCM 15050) which consisted of a *phaC*, *phaA*, *phaB* and *phaR* were successfully cloned. The heterologous expression of *Burkholderia* sp. PHA synthase gene in *C*. *necator* PHB¯4 showed that the PHA synthase was capable of polymerizing 4HB monomer. The biosynthesis results also suggested *Burkholderia* sp. PHA synthase preference for 4HB-CoA as PHA with high 4HB composition (87 mol%) was synthesized by transformant *C*. *necator* harbouring the synthase. PHA synthase activity assay of wild-type *Burkholderia* sp. and transformant *C*. *necator* indicated that these two strains exhibited activities that are comparable and in a similar range with *C*. *necator* H16. More efforts to analyze the substrate specificity of PHA synthase in various hosts need to be done in order to understand fully the potential of the synthase.

## Competing interests

The authors declare that they have no competing interests.
